# High-Throughput 3D Tumor Spheroid Array Platform for Evaluating Sensitivity of Proton-Drug Combinations

**DOI:** 10.3390/ijms23020587

**Published:** 2022-01-06

**Authors:** Dong Woo Lee, Jung Eun Kim, Ga-Haeng Lee, Arang Son, Hee Chul Park, Dongryul Oh, Kwanghyun Jo, Changhoon Choi

**Affiliations:** 1 Department of Biomedical Engineering, Konyang University, Daejeon 35365, Korea; dw2010.lee@gmail.com; 2 Medical & Bio Decision (MBD), Suwon 16229, Korea; kje0926@mbdbiotech.com; 3 Department of Radiation Oncology, Samsung Medical Center, Seoul 06351, Korea; vitamin_milk@naver.com (G.-H.L.); onlyshohow@naver.com (A.S.); hee.ro.park@gmail.com (H.C.P.); dongryuloh@gmail.com (D.O.); 4 Department of Radiation Oncology, Sungkyunkwan University School of Medicine, Seoul 06351, Korea

**Keywords:** proton beam therapy, tumor spheroids, high-throughput screening platform, olaparib, combination index

## Abstract

Proton beam therapy (PBT) is a critical treatment modality for head and neck squamous cell carcinoma (HNSCC). However, not much is known about drug combinations that may improve the efficacy of PBT. This study aimed to test the feasibility of a three-dimensional (3D) tumor-spheroid-based high-throughput screening platform that could assess cellular sensitivity against PBT. Spheroids of two HNSCC cell lines—Fadu and Cal27—cultured with a mixture of Matrigel were arrayed on a 384-pillar/well plate, followed by exposure to graded doses of protons or targeted drugs including olaparib at various concentrations. Calcein staining of HNSCC spheroids revealed a dose-dependent decrease in cell viability for proton irradiation or multiple targeted drugs, and provided quantitative data that discriminated the sensitivity between the two HNSCC cell lines. The combined effect of protons and olaparib was assessed by calculating the combination index from the survival rates of 4 × 4 matrices, showing that Cal27 spheroids had greater synergy with olaparib than Fadu spheroids. In contrast, adavosertib did not synergize with protons in both spheroids. Taken together, we demonstrated that the 3D pillar/well array platform was a useful tool that provided rapid, quantitative data for evaluating sensitivity to PBT and drug combinations. Our results further supported that administration of the combination of PBT and olaparib may be an effective treatment strategy for HNSCC patients.

## 1. Introduction

As a curative-intent treatment or an adjuvant to surgery, radiation therapy (RT) has been employed in the treatment of head and neck cancer (HNC) [1,2]. Proton beam therapy (PBT) is the most popular type of particle-based RT. Compared to conventional photon RT, PBT offers dosimetric advantages due to a distinct physical property called the “Bragg peak”. In HNC treatment, PBT can spare critical organs, including salivary glands, eyes, and oral cavity structures, from unnecessary exposure to radiation while delivering high radiation doses to tumor sites [3,4,5]. Mounting evidence suggests that PBT provides excellent locoregional control with less acute toxicities than photon RT in HNC treatment. Nevertheless, only a few preclinical studies have compared the biological advantages of PBT over photon RT in HNC models [6,7].

The difference in the biological impact of PBT over photon RT is expressed as the relative biological effectiveness (RBE) [8]. The RBE of PBT is defined as the ratio of the proton doses to photon doses that yield the same biological endpoints, such as clonogenic survival and jejunum crypt regeneration [8,9,10]. In the clinic, a constant RBE value of 1.1 is used regardless of tumor intra- or inter-heterogeneity. Many efforts have been devoted to the investigation of biomarkers or radiosensitizers that potentially enhance the efficacy of PBT. Recent findings from our and other researchers have shown that several targeted drugs increased the sensitivity of various cancer cells to proton irradiation over photon irradiation, resulting in an increase in the RBE [11,12,13,14,15,16]. For instance, a cell cycle checkpoint inhibitor [11], a histone deacetylase inhibitor [14], a PARP inhibitor [16], and cisplatin [17] increased proton sensitivity across many types of cancer cells. Several genes involved in DNA damage repair pathways, such as RAD51 and FANCD2, have been proposed as putative biomarkers related to proton sensitivity [13,18,19].

In 2015, The Cancer Genome Atlas (TCGA) consortium reported the largest set of genomic data of head and neck squamous cell carcinoma (HNSCC), providing a comprehensive landscape of the genomic alterations, including druggable targets [20]. Regarding RT response, human papilloma virus (HPV) infection is strongly associated with favorable responses, whereas mutations in TP53 in HPV-negative HNSCC confer radioresistance [21]. Aberrant signaling of receptor tyrosine kinases, including epidermal growth factor receptor and its downstream, and upregulation of DNA damage repair proteins such as Ku80 and poly (ADP-ribose) polymerase 1 (PARP1) contribute to radioresistance in HNSCC [22]. Besides these intrinsic factors, tumor hypoxia and immune suppressive microenvironment are key extrinsic factors that cause a failure of RT. Hypoxia-activated prodrugs or immunotherapy hold great promise to overcome these hurdles and improve treatment outcome [23,24].

Despite the increasing use of PBT for HNSCC treatment, there are few systemic approaches to facilitate the identification of targeted drugs that synergistically interact with PBT. In this study, we aimed to test the feasibility of a three-dimensional (3D) spheroid-culture-based high-throughput screening (HTS) platform to evaluate the response of HNSCC cells to PBT. For more robust screening, we designed a proton treatment plan that allowed the precise delivery of four graded proton doses onto a single 384-well plate using a pencil beam scanning mode. Using a newly designed HTS platform, we screened synergistic combinations of anticancer drugs and PBT using two human HNSCC cell lines.

## 2. Results

### 2.1. Setup of the 3D-Based HTS Platform and Its Dosimetric Analysis

**Figure 1:**
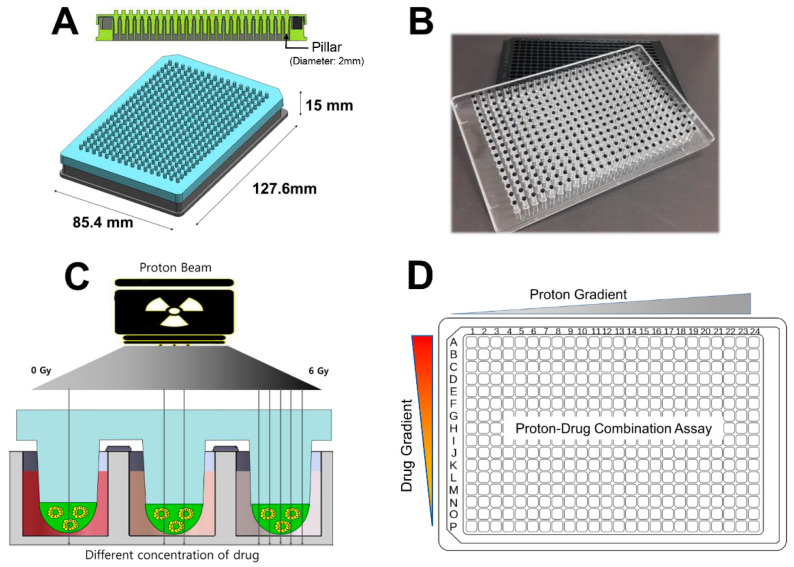
The 3D spheroid-based 384-pillar/well platform used for this study: (**A**) schematic view of the 384-pillar/well platform; (**B**) fabricated platform; (**C**) schematic view of proton–drug combination screening; (**D**) layout of the proton–drug combination.

Various HTS technologies using 3D spheroid-based multiwell pillars have been developed to determine therapeutic responses in cancer [25]. We utilized this technology to develop a new system for concomitantly measuring the combined effects of proton radiation and anticancer drugs. For this, we adopted the 384-pillar/well platform (Figure 1A,B), which was proven to be suitable for drug screening under 3D culture conditions [25]. Proton beams with a spread-out Bragg peak (SOBP) of 30 mm width were perpendicularly delivered to the 384-pillar/well plate (Figure 1C). The plate was mapped to have a layout that increased the proton dose in the transverse direction and the drug dose in the longitudinal direction for evaluating proton–drug combinations (Figure 1D).

**Figure 2:**
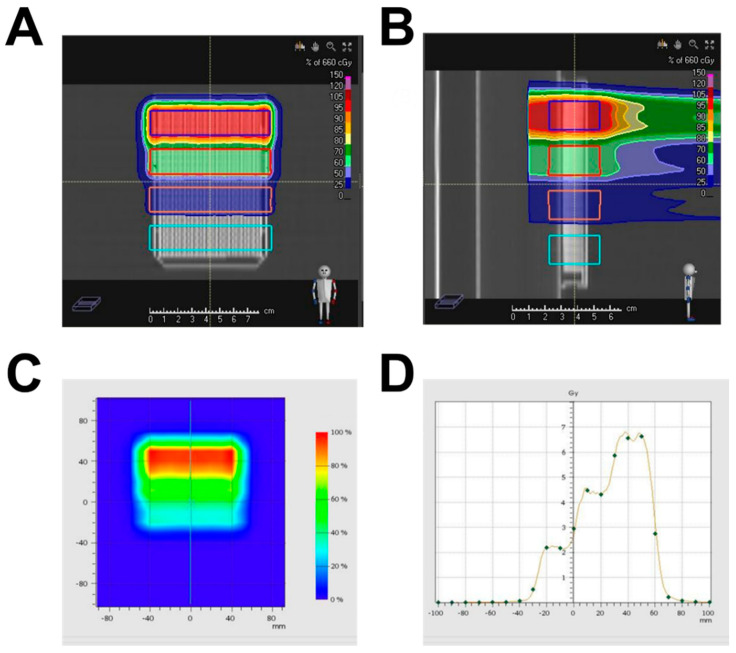
Dose distribution of the 4-step cell irradiation plan. Homogeneous dose distributions were achieved by each plateau, and delivered doses decreased in a stepwise manner. (**A**) Coronal view; (**B**) sagittal view. (**C**) Delivered doses were measured by 2D ion chamber array. (**D**) One-dimensional dose distribution of plan and measurement were plotted. The measured dose points were spaced at 1 cm intervals, and are denoted as dots. The solid line indicates the plan dose.

A treatment plan was created to deliver four doses of proton beams ranging from 0 to 6 Gy onto a 384-well plate in a stepwise manner. Using computed tomography (CT) images of the 384-well plate, the entire plate area was divided into four target regions. Each region was assigned a graded dose of 0, 2, 4, or 6 Gy (Figure 2A,B). Each dose area was planned to have a plateau region covering at least three rows of wells, as indicated by the solid lines in Figure 2A,B. The calculated proton-dose distribution was measured and verified using an ionization chamber array (Figure 2C,D).

### 2.2. HNSCC Cell Sensitivity to Proton Irradiation Using the 3D-Based HTS Platform

**Figure 3:**
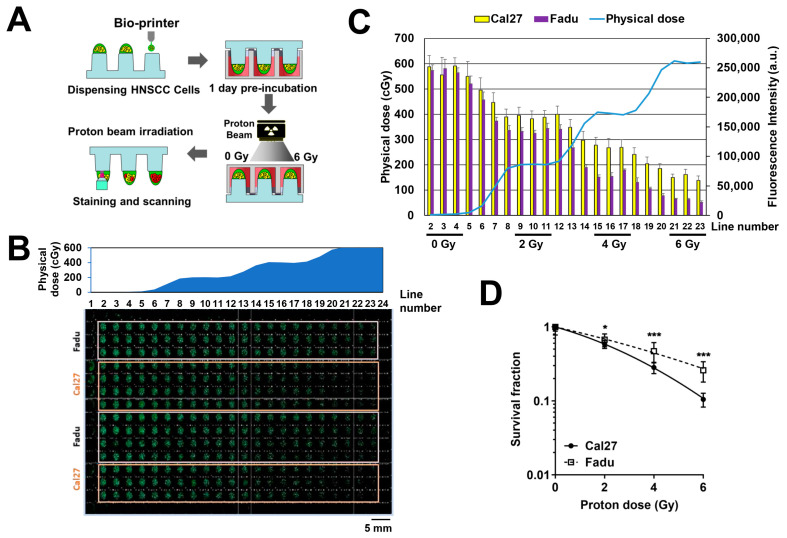
An HTS platform for determining the cellular sensitivity against proton therapy. (**A**) Scheme of a 3D tumor spheroid platform for measuring the sensitivity to proton irradiation. (**B**) Layout of a 384-pillar/well plate containing Fadu and Cal27 spheroids for proton treatment. Representative fluorescent image indicating viable Fadu and Cal27 spheroids after proton irradiation. White and orange boxes indicate samples used for quantitative analysis. The upper graph represents the physical dose profile of protons on the plate. (**C**) Quantification of fluorescence intensity of Fadu and Cal27 spheroids stained with Calcein AM along each vertical line in a 384-well plate. (**D**) Survival curves of Fadu and Cal27 spheroids receiving the indicated doses of protons. Survival fraction was calculated as the ratio of fluorescence intensity at the indicated proton dose to that at 0 Gy. Data are mean ± S.D. (*n* = 21) from two independent experiments. * *p* < 0.05; *** *p* < 0.001.

Next, we evaluated whether the 3D-based HTS platform measured the dose-dependent sensitivity of HNSCC cells to proton irradiation. Fadu and Cal27 cells were suspended in a matrix consisting of 0.5% alginate and 50% Matrigel, and the mixture was automatically spotted on top of the pillars (Figure 3A). After 1 day of preincubation, the pillar/well plates were irradiated with proton beams using the prescribed treatment plan. After 7 days of incubation, the spheroids were stained with Calcein AM, a membrane-permeable live-cell labelling dye. Fluorescent images of the spheroids stained with Calcein AM in an entire 384-well plate showed that green fluorescence signals decreased with an increasing proton dose from left to right (Figure 3B). Quantitative data confirmed that fluorescence intensity was inversely correlated with the physical dose (Figure 3C). Data were acquired from flat areas with proton doses of 0, 2, 4, and 6 Gy, which corresponded to three consecutive rows (Figure 3C). Dose–response curves showed that Fadu cells were more radioresistant to Cal27 under 3D-culture conditions (Figure 3D).

### 2.3. HNSCC Cell Sensitivity to Anticancer Drugs Using the 3D-Based HTS Platform

**Figure 4:**
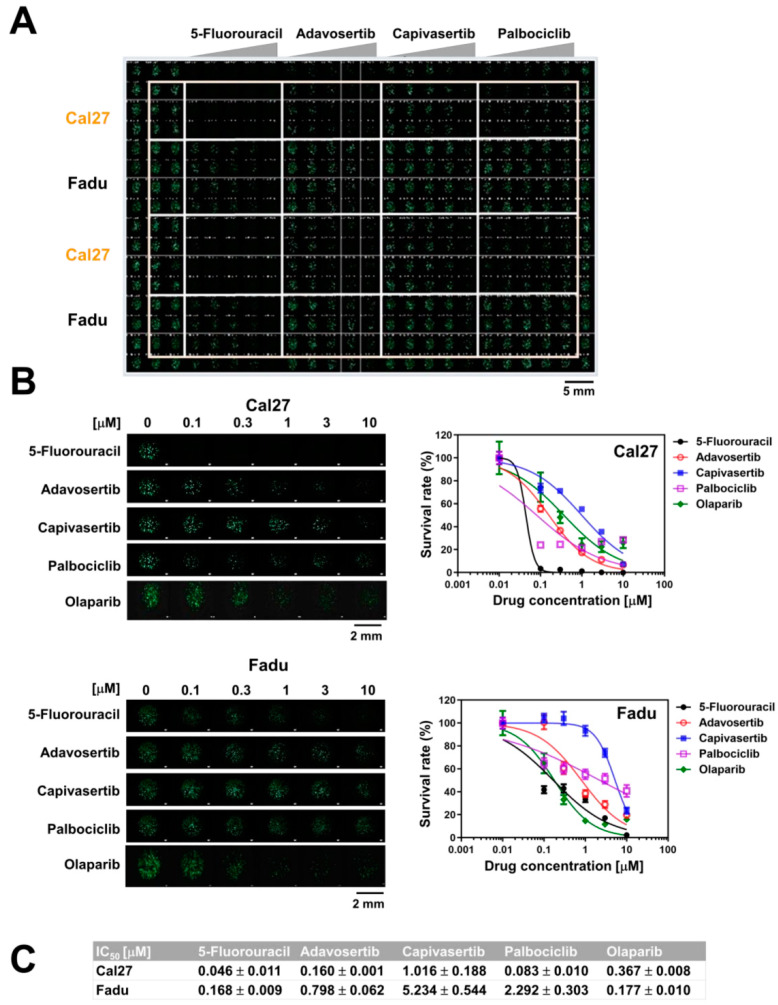
Measurement of the drug sensitivity in HNSCC spheroids using the 3D-based HTS platform. (**A**) Layout of a 384-pillar/well plate containing Cal27 and Fadu spheroids for different drug treatments. Representative fluorescence image was captured after 7 days of incubation with drugs. (**B**) Comparison of sensitivity of Cal27 and Fadu spheroids in response to five anticancer drugs at the indicated concentrations. Left, representative fluorescent images of Cal27 and Fadu spheroids showing different responses to the drugs. Right, Dose–response curves of Cal27 and Fadu spheroids. (**C**) IC_50_ values of five anticancer drugs on Cal27 and Fadu spheroids. Data are mean ± S.D. (*n* = 7) from two independent experiments.

We utilized the 3D-based HTS platform to determine the sensitivity of the two HNSCC cell lines to various anticancer drugs. Spheroids of Fadu and Cal27 cells were allowed to grow onto 384 pillars and were then exposed to five anticancer drugs at a concentration of 0 to 10 µM for 7 days. The dose–response curves and IC_50_ values were calculated from the fluorescence intensity values of the scanned images. Scanned images of spheroids treated with each drug showed a dose-dependent decrease in the number of Calcein-AM-stained spheroids (Figure 4A). In addition, 5-fluorouracil, a chemotherapeutic drug for HNSCC, dramatically decreased the viability of spheroids of both Fadu and Cal27 (IC_50_ = 0.168 and 0.044, respectively; Figure 4B,C). Compared with Fadu, Cal27 spheroids were more sensitive to other anticancer drugs, including adavosertib (Wee1 inhibitor), capivasertib (AKT inhibitor), and palbociclib (CDK4/6 inhibitor), but not olaparib (PARP inhibitor). These data suggested that our 3D-based HTS platform was useful for comparing the effects of multiple anticancer drugs on spheroid growth, as well as proton beams, in a quantitative manner.

### 2.4. Combination Effect of Proton Irradiation and Olaparib in the 3D-Based HTS Platform

**Figure 5:**
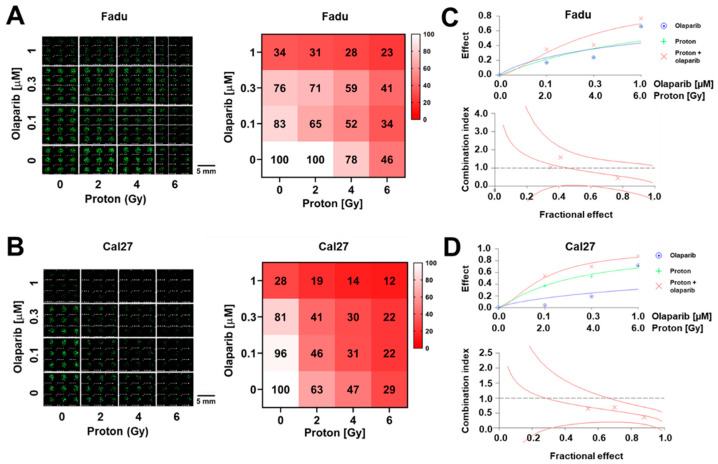
Measurement of the combination effect of proton and olaparib on the growth of HNSCC spheroids using the 3D-based HTS platform. (**A**,**B**) Sensitivity of Fadu (**A**) and Cal27 (**B**) spheroids to combination therapy with proton and olaparib. Left, fluorescence images of 384-pillar/well plates containing Fadu and Cal27 spheroids treated with indicated doses of protons and olaparib. Right, 4 × 4 matrices showing survival rates of Fadu and Cal27 spheroids for different combination treatment conditions. Color scale represents relative survival rates. (**C**,**D**) Combination index (CI) values of Fadu (**C**) and Cal27 (**D**) at indicated doses of proton and olaparib. Top, dose–effect curves. Bottom, CI values calculated by CalcuSyn software (Biosoft, Ferguson, MO, USA). The X-marks represent the CI values of the combination treatment groups. The middle curve line represents the simulated CI values of the combination treatment groups surrounded by two lines of algebraic estimations of the 95% confidence intervals.

Olaparib is a well-known radiosensitizer in many types of cancers, including HNSCC. To confirm the utility of the 3D-based HTS platform for proton sensitizer screening, we tested the combined effects of protons and olaparib. Spheroids of Fadu and Cal27 were allowed to grow onto 384 pillars for a day, followed by treatment with olaparib and protons on the same day. After 7 days of incubation, the spheroids were stained with Calcein AM. Scanned fluorescence images showed that the number of Calcein-stained spheroids of both Fadu and Cal27 decreased with increasing doses of olaparib and protons (Figure 5A,B, right). The scanned image was converted into a 4 × 4 matrix of survival rates; each element represented the relative survival rates calculated from the averaged fluorescence intensity of spheroids grown under the indicated treatment conditions (Figure 5A,B, left). Analyses of the survival data matrix showed that olaparib had a synergistic effect with protons in both HNSCC spheroids, which was judged by calculation of the combination index (CI) using CalcuSyn software; the synergism was more evident in Cal27 than Fadu (Figure 5C,D). We also tested another combination of protons and adavosertib in both Fadu and Cal27 spheroids, and found that there was no additive effect (Figure S1).

**Figure 6:**
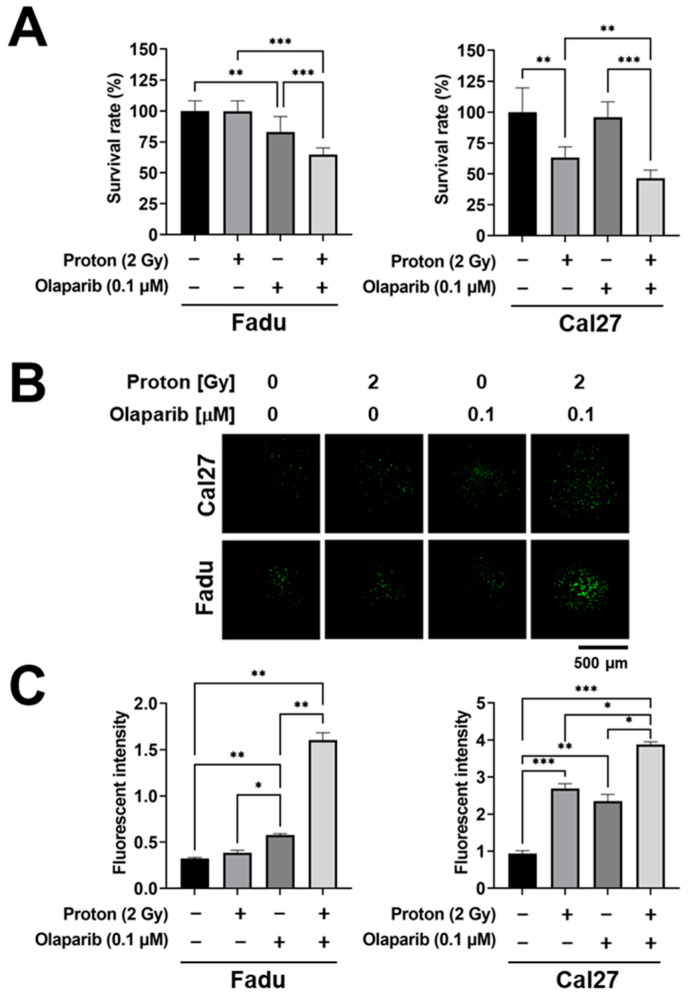
Effect of combination treatment with proton and olaparib on the apoptosis of HNSCC spheroids. (**A**) Comparison of survival rates of Fadu and Cal27 treated with 2 Gy of proton and 0.1 µM olaparib. Data are mean ± S.D. (*n* = 9). (**B**) Apoptotic cell death induced by the combination treatment with protons and olaparib as determined by caspase-3/7 detection assay. Representative fluorescent images of Cal27 and Fadu spheroids related to caspase-3/7 activity. (**C**) Quantification of fluorescence intensity showing an increase in apoptotic death of spheroids by the combination with proton and olaparib. Data are mean ± S.D. (*n* = 3). * *p* < 0.05; ** *p* < 0.01; *** *p* < 0.001.

Next, we investigated how the combination of olaparib sensitized 3D spheroids to proton irradiation. In the survival data matrix, we found that both Cal27 and Fadu spheroids had an additive effect on the reduction of survival rates upon treatment with 0.1 µM olaparib and 2 Gy of protons, a conventional daily fraction dose of radiotherapy (*p* < 0.001; Figure 6A). A caspase-3/7 staining assay revealed that olaparib alone increased apoptosis in both Cal27 and Fadu spheroids (*p* < 0.01; Figure 6B,C). Protons also increased apoptosis of proton-sensitive Cal27 spheroids, but not Fadu spheroids. Cotreatment with olaparib significantly enhanced the expression of caspase-3/7 in Fadu spheroids (*p* < 0.01) and Cal27 spheroids (*p* < 0.05; Figure 6B,C). These data validated the compatibility of our 3D-based HTS platform for screening proton radiosensitizers with mechanistic investigations through quantitative analysis.

## 3. Discussion

HNSCC is the sixth most common cancer worldwide, and photon-based chemoradiation is the current standard of care. The number of HNSCC patients receiving proton beam therapy is rapidly increasing, but how the biological influence of proton therapy differs from that of photon-based therapy in HNSCC remains unclear. Recently, several comparative studies have demonstrated the difference in biological effectiveness of proton over photon radiotherapy, and revealed the underlying mechanisms. Reverse-phase protein array analysis showed that a single 4 Gy dose of protons increased the expression of DNA damage repair (DDR), cell cycle arrest, and antiproliferation to a greater extent than X-rays [26,27]. Protons caused more persistent DNA double-strand breaks (DSBs) in HNSCC cells than protons, even though the related DSB repair mechanisms remained undetermined [26,27]. Regarding cell death, protons produced higher proportions of HNSCC cells undergoing mitotic catastrophe and senescence with only limited apoptosis [6]. It is likely that protons are less immunosuppressive than photons because of the normal tissue-sparing effect. These fundamental differences between the two radiation modalities may affect the response to drugs targeting the DDR signaling pathway, such as PARP inhibitors [15,28]. The current study aimed to develop an HTS platform to screen drugs to enhance proton sensitivity.

Currently, 3D cell culture systems are widely accepted as the most effective research platform for determining drug responses in vitro. They may better reflect in vivo tumor environments, such as gradients of nutrients, oxygen, and pH, than 2D monolayer cultures, although they cannot fully simulate such in vivo features. Cancer cells cultured in 3D are more resistant to radiation than cells cultured in 2D [29,30]. Moreover, a 3D spheroid culture in a 384-well format enables researchers to perform HTS or imaging-based high content screening (HCS). However, most studies for measuring HTS-based radiosensitivity have adopted a method to deliver a single radiation dose to a multiwell plate and measure relative radiation responses compared to untreated control plates. This is highly efficient, but plate-to-plate variation may cause problems with accuracy and reproducibility. To avoid these issues and determine the proton–dose response more precisely, we utilized scanning beamlets of protons based on spot-scanning technology, which allowed us to paint a single 384-well plate with four graded proton doses (Figure 2). In the current study, two human HNSCC cells were spotted onto a single plate due to the availability of only two spot nozzles (Figure 3B), but the process can be technically improved with multiple nozzles. Proton–dose response curves were successfully obtained, showing that Fadu spheroids were more resistant than Cal27 spheroids.

Our screening using the HTS platform confirmed that olaparib—a PARP inhibitor—acted as a proton radiosensitizer in HNSCC cells (Figure 4). A previous study by Hirai et al. showed that olaparib sensitized two human cancer cell lines, A549 and MIA PaCa-2, to proton beam irradiation with enhanced γH2AX expression and G2/M arrest [31]. Olaparib also sensitized radioresistant HNSCC Fadu cells via PARP1 inhibition [32]. Wang et al. showed that another PARP inhibitor, niraparib, increased the sensitivity of HNSCC cells to both photon and proton irradiation [15]. In HPV-negative and HPV-positive cell lines, niraparib treatment increased proton RBE by 3% and 10%, respectively. Olaparib potentiated fractionated proton irradiation in esophageal cancer cells [16]. Our caspase-3/7 staining data revealed that olaparib plus protons activated caspase cascades in two HNSCC cell lines, thereby inducing apoptotic cell death (Figure 6C).

A feature of our HTS platform is that it facilitates the measurement of the synergy between two different treatments through two-dimensional analysis. It seemed that olaparib sensitized both Cal27 and Fadu cells to proton irradiation, but the CI index evaluation showed that the synergism between protons and olaparib was more prominent in Cal27 cells than in Fadu cells. Accumulating evidence has shown that the radiosensitization of PARP inhibitors is dependent on defects in homologous recombination (HR) repair [33]. Thus, it would be interesting to test whether there was a difference in HR repair proficiency between Cal27 and Fadu cells. Unlike olaparib, adavosertib—the Wee1 inhibitor—did not show any additive effect with proton irradiation in either Cal27 or Fadu cells (Figure S1). G2-checkpoint-targeting agents such as Wee1 inhibitors are more effective in combination with radiation in HPV-positive than HPV-negative cells, and dual targeting of Chk1 and Wee1 greatly enhanced radiosensitization regardless of HPV status [34,35]. Thus, it is assumed that Adavosertib plus Chk1 inhibitors may show better synergy with protons. Theoretically, the treatment sequence could influence any combination data, including those without synergism of adavosertib and protons, and requires further validation. Recent studies have recommended that testing the drug efficacy in a single spheroid is desirable, because the data reproducibility of 3D models depends on morphological parameters such as volume and shape [36,37]. Thus, further morphological analysis of individual spheroids grown on a single pillar could provide valuable information.

Radiotherapy remains the mainstay of treatment for patients with HNSCC, but requires further improvement. A variety of molecularly targeted agents, such as DDR inhibitors, cell cycle blockers, and epidermal growth factor receptor inhibitors, have been tested in the presence of X-rays [22]. However, whether they exert a synergistic antitumor effect with protons remains largely unknown. Meerz et al. showed that inhibition of ATM, DNA-PK, and PARP sensitized 3D-cultured HNSCC cells to both proton and photon irradiation [38]. Cells were cultured in a 96-well plate and treated with different inhibitors at a single concentration. In contrast, our HTS platform allowed the measurement of the sensitizing effect for a wide range of drug/proton doses, enabling the detection of subtle differences in combination efficacy. For further validation, in vivo studies using laboratory animal models (e.g., patient-derived xenograft models for the same cell line) need to be performed. Recently, patient-derived organoids (PDOs) have attracted attention as tools to predict the sensitivity of patients with rectal cancer and HNSCC to chemoradiation [39,40]. Application of PDO cultures to our HTS platform and integration of patient information will open up new avenues for precision proton therapy.

## 4. Materials and Methods

### 4.1. Cell Cultures

Human head and neck squamous carcinoma Fadu and Cal27 cell lines were obtained from the American Type Culture Collection (ATCC, Manassas, VA, USA). Fadu cells were cultured in Eagle’s minimum essential medium (EMEM, Gibco, Carlsbad, CA, USA) supplemented with 10% fetal bovine serum (FBS, Gibco) and antibiotics, and Cal27 cells were cultured in Dulbecco’s modified Eagle’s medium (DMEM, Gibco) supplemented with 10% FBS and antibiotics. Cultures were maintained in a humidified incubator with 5% CO_2_ at 37 °C. Before use, all cells were authenticated by short tandem repeat (STR) profiling and tested for mycoplasma contamination.

### 4.2. 3D Cell Printing

Fadu and Cal27 spheroids were prepared on the micropillar chip as previously described [25]. Briefly, 400 cells in 1 µL of 0.5% alginate and 50% Matrigel (*w*/*w*) were automatically dispensed onto a micropillar chip using ASFA Spotter ST (Medical & Bio Decision, Suwon, Korea). After 1 min of gelation, the micropillar chips containing the 3D spheroids were placed in a 384-microwell plate containing growth media. The next day, micropillar chips were exposed to radiation or transferred to a new 384-microwell plate containing anticancer drugs.

### 4.3. Cell Viability and Apoptosis Measurement

Cell viability was determined by staining with Calcein AM (Invitrogen, Carlsbad, CA, USA) according to the manufacturer’s instructions. Briefly, 3D spheroids were incubated with Calcein AM staining solution (0.5 µM Calcein AM, 140 mM NaCl, and 20 mM CaCl_2_) for 1 h and then washed twice with phosphate-buffered saline (PBS) for 20 min. Dried alginate spots were scanned with an automatic optical fluorescence scanner (ASFA Scanner HE, Medical & Bio Decision). The microscope in the scanner automatically focused on the cell spots by moving in the z-direction, and took 384 individual pictures from a single stained pillar/well plate at 4× magnification. The 384 pictures of the cell spots were then consolidated into a single JPEG image for data analysis. The scanned images were analyzed using CellAnalyzer version 1.0 (Medical & Bio Decision).

Apoptosis was determined by using CellEvent^TM^ Caspase-3/7 green detection reagent (Thermo Fisher Scientific, Waltham, MA, USA) according to the manufacturer’s instructions. Briefly, after 3 days of treatment with protons and/or olaparib, spheroids were incubated with caspase-3/7 reagent for 1 h at 37 °C. The fluorescence images were acquired using an ASFA optical fluorescence scanner, and were analyzed using ImageJ software version 1.53e (National Institutes of Health, Bethesda, MD, USA).

### 4.4. Dose–Response Curves and IC_50_ Calculation

Cell viability values were normalized to the corresponding control wells (no drug treatment). The sigmoidal dose–response curves (variable slope) and IC_50_ values (i.e., concentration of the compound in which 50% of cell growth was inhibited) were obtained using the following equation:

(1) $$Y=Bottom+\left[\frac{Top-Bottom}{1+10^{(\log\mathrm{IC}50-X)\times n_{H}}}\right]$$

where IC_50_ is the midpoint of the curve, *n_H_* is the hill slope, *X* is the logarithm of the compound concentration, and *Y* is the response (cell viability). The Prism software set the bottom as zero and the top as 100% when the data were fitted to a curve.

### 4.5. Combination Effect Analysis

A combination of drug and proton treatments using 384-pillar plates was performed by dispensing compounds into a 384-well plate and treating protons, as shown in Figure 1D. The two-parameter combination effect was analyzed using the median-effect methods of Chou and Talalay with CalcuSyn software ver. 2.1.1 (Biosoft, Ferguson, MO, USA) [41,42]. CI < 1, CI = 1, and CI > 1 indicated synergy, additive effect, and antagonism, respectively.

### 4.6. Irradiation

CT images were acquired to determine the exact geometry of the 384-multiwell cell culture plate. The entire plate area was divided into four target regions: planning target volumes (PTVs) 1, 2, 3, and 4. The PTVs were delineated in the CT image, and each PTV was separated by 10 mm intervals (Figure 1A). A pillar structure with a diameter of 2 mm was positioned inside the individual well filled with the medium. The CT density was overridden with water density because the density of the medium was approximately equal to that of water.

Treatment planning was performed using Raystation v6.B (Raysearch AB, Stockholm, Sweden) with intensity modulation. The computation was performed using a Monte Carlo dose engine. Graded dose distribution was achieved by assigning different values to each PTV: 0 Gy to PTV1, 2 Gy to PTV2, 4 Gy PTV3, and 6 Gy to PTV4. Considering the difficulty in making 0 Gy in PTV1 by intensity modulation, the aperture was used to completely block the proton beams to PTV1. The graded dose distribution of the treatment plan is illustrated in Figure 2A,B. Each plateau region included at least three rows of wells, and the number of data points was 48 (16 × 3) for each PTV. To guarantee stability of the flat dose in PTVs, the treatment plan was designed to generate a spread-out Bragg peak (SOBP) with a width of 30 mm. The highest energy of the proton beam was 187.6 MeV, and eight energy layers were used.

### 4.7. Dosimetry

Proton beam irradiation was performed using an SHI proton system (Sumitomo Heavy Industry Ltd., Tokyo, Japan). The absorbed dose in the four flat regions was measured using a PPC05 ion chamber (IBA, Louvain la Neuve, Belgium). The measurement was performed at mid-SOBP, 203 mm, for a 187.6 MeV proton beam with a 30 mm width of SOBP. The measurement points were selected at the center of each PTV. The absorbed dose differences between the treatment plan and measurements are summarized in Table 1. The two-dimensional (2D) dose distribution was measured using OCTAVIUS detector 729 arrays (PTW, Freiburg, Germany) with 729 vented ion chambers. The gamma passing rate between the measured and planned doses was 100% with a 2 mm distance to agreement and a 2% dose difference.

### 4.8. Statistics

Statistical analysis was performed using GraphPad Prism 9.0 (GraphPad, San Diego, CA, USA). The IC_50_ values were estimated from the dose–response curves using nonlinear regression analysis. Data are expressed as mean ± standard deviation (SD) from at least two independent experiments. The *p* values were calculated using one-way analysis of variance (ANOVA) with Tukey’s multiple comparison correction. Statistical significance was set at *p* < 0.05.

## Figures and Tables

**Table 1:** Dose differences between plan and measurement by ion chamber.

	Plan	Measurement	Difference (%)
PTV2	203.6 cGy	205.5 cGy	0.9%
PTV3	397.5 cGy	398.7 cGy	0.3%
PTV4	611.4 cGy	603.4 cGy	−1.3%

## Data Availability

Not applicable.

## Supplementary Materials

The following are available online at https://www.mdpi.com/article/10.3390/ijms23020587/s1.
