# Supplementary material for: High-Throughput 3D Tumor Spheroid Array Platform for Evaluating Sensitivity of Proton-Drug Combinations
Source: Int J Mol Sci. 2022 Jan 6;23(2):587. doi: 10.3390/ijms23020587 (PMC8775525; doi:10.3390/ijms23020587)
Supplement: Supplementary file 1 [file ijms-23-00587-s001.zip › ijms-1507540-supplementary.pdf]

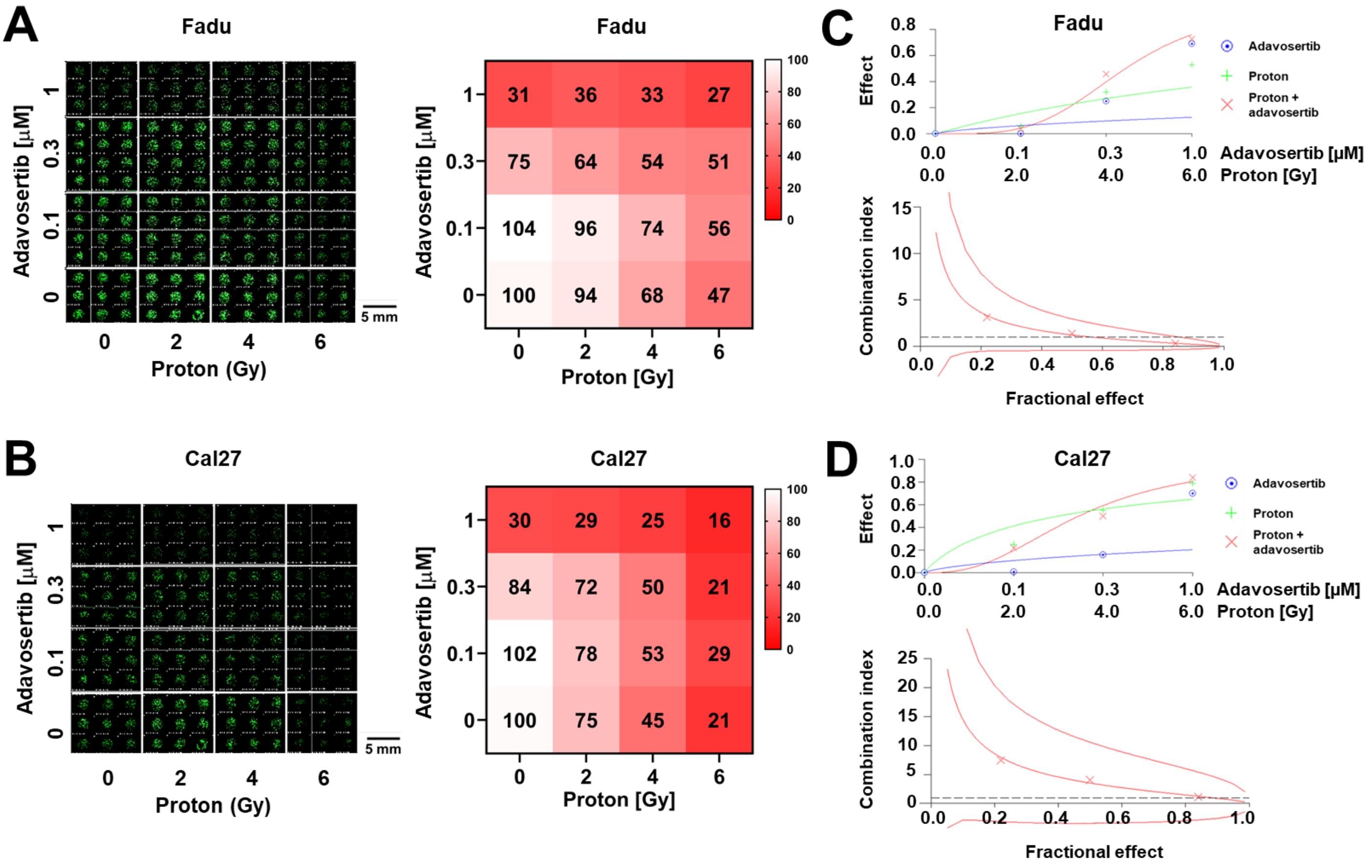

Figure S1. Combination effect of proton and adavosertib on the growth of HNSCC spheroids. (A and B) Sensitivity of Fadu (A) and Cal27 (B) spheroids to combination therapy with proton and adavosertib. Left, fluorescence images of 384 pillar/well plates containing Fadu and Cal27 spheroids treated with indicated doses of protons and adavosertib. Right, 4-by-4 matrices showing survival rates of Fadu and Cal27 spheroids for different combi-nation treatment conditions. Color scale represents relative survival rates. (C and D) Combination index (CI) values of Fadu (C) and Cal27 (D) at indicated doses of proton and adavosertib. Upper, dose-effect curves. Bottom, CI values calculated by CalcuSyn software. The X-marks represent the CI values of the combination treatment groups. The middle curve line represents the simulated CI values of the combination treatment groups surrounded by two lines of algebraic estimations of the 95% confidence intervals.
